# P-367. Pharmacists’ Decisions on Switching ART to Address Patient Dissatisfaction With Daily Oral Therapy: Results From an Educational Program

**DOI:** 10.1093/ofid/ofaf695.585

**Published:** 2026-01-11

**Authors:** Logan Van Ravenswaay, Edward King, Jenny Schulz

**Affiliations:** Clinical Education Alliance, Loami, Illinois; Clinical Care Options, LLC, Reston, VA; Clinical Care Options, Atlanta, GA

## Abstract

**Background:**

Optimizing antiretroviral therapy (ART) requires healthcare professionals to regularly reappraise whether each treated person living with HIV is receiving the regimen most suited to their individual needs, including people who have achieved virologic suppression on their current regimen but may be dissatisfied with aspects that could be addressed by switching to a different regimen. Pharmacists have a key role in decision-making about ART and the potential to optimize care.

**Methods:**

As part of a CME program comprising in-person meetings and webinars led by expert faculty, we assessed baseline practice and intended practice change using preactivity and postactivity assessments. Here, we focus on responses to a case-based question that explored whether a person who is virologically suppressed on a contemporary daily oral regimen but complains that taking a pill every day reminds him of his HIV infection should be considered a candidate for switching to long-acting cabotegravir plus rilpivirine (LA CAB + RPV), which expert faculty determined to be the optimal management strategy. We analyzed responses received from pharmacists between March 1, 2024, and March 4, 2025.

**Results:**

718 pharmacists provided either a pretest or a posttest response, with 557 providing both. 286 (39.8%) pharmacists self-identified as specializing in HIV/ID. Among all respondents (unmatched) at pretest, 52% selected the optimal strategy of considering a switch to LA CAB + RPV, increasing to 78% at posttest following education (P < .001). Responses were similar among the subset of respondents with matched responses (54% vs 79%; P < .001). The percentage that would switch to a different oral regimen (which would not address the person’s dissatisfaction with daily oral ART) decreased from 19% to 11%. The percentage that would not consider any switch decreased from 29% to 12%. Differences in responses by demographic and geographic variables will be reported.

**Conclusion:**

At baseline, most pharmacists would not consider switching ART in a person who expressed dissatisfaction with daily oral therapy. A subset would consider an inappropriate switch to a different daily oral regimen. Following education, most respondents would consider switching to LA CAB + RPV in this setting.

**Disclosures:**

Logan Van Ravenswaay, PharmD, AbbVie: Stocks/Bonds (Public Company)|Bristol-Myers Squibb: Stocks/Bonds (Public Company)|Lilly: Stocks/Bonds (Public Company)|Novo Nordisk: Stocks/Bonds (Public Company)

